# *Babesia microti* Infection Inhibits Melanoma Growth by Activating Macrophages in Mice

**DOI:** 10.3389/fmicb.2022.862894

**Published:** 2022-06-22

**Authors:** Xiang Shu, Zheng Nie, Wanxin Luo, Yaxin Zheng, Zhen Han, Hongyan Zhang, Yingjun Xia, Han Deng, Fangjie Li, Sen Wang, Junlong Zhao, Lan He

**Affiliations:** ^1^State Key Laboratory of Agricultural Microbiology, College of Veterinary Medicine, Huazhong Agricultural University, Wuhan, China; ^2^Key Laboratory of Animal Epidemical Disease and Infectious Zoonoses, Ministry of Agriculture, Huazhong Agricultural University, Wuhan, China

**Keywords:** *Babesia microti*, melanoma, antitumor, immunotherapy, macrophage polarization

## Abstract

*Babesia microti* is an obligate intraerythrocytic protozoan transmitted by *an Ixodes* tick. Infections caused by protozoa, including *Plasmodium yoelii* and *Toxoplasma gondii*, are shown to inhibit tumor development by activating immune responses. Th1 immune response and macrophages not only are essential key factors in *Babesia* infection control but also play an important role in regulating tumor development. In this study, we investigated the effects of *B. microti* infection on melanoma in tumor-bearing mice. The results showed that *B. microti* infection could inhibit the growth of melanoma, significantly enlarge the spleen size (*p* ≤ 0.0001), and increase the survival period (over 7 days) of tumor-bearing mice. Mouse spleen immune cell analysis revealed that *B. microti*-infected tumor-bearing mice could increase the number of macrophages and CD4+ T cells, as well as the proportion of CD4+ T cells and M1 macrophages in the tumor. Immunohistochemical assays showed that *B. microti* infection could inhibit tumor angiogenesis (*p* ≤ 0.0032). Meanwhile, both *B. microti*-infected erythrocytes and culture supernatant were observed to significantly (*p* ≤ 0.0021) induce the mRNA expression of iNOS, IL-6, and TNF-α in macrophages. Moreover, *B. microti* culture supernatant could also repolarize IL-4-induced M2 macrophages to the M1 type. Overall, *B. microti* exerted antitumor effects by stimulating the immune system of tumor-bearing mice and inducing the polarization of immunosuppressive M2 macrophages to pro-inflammatory M1 macrophages.

## Introduction

*Babesia microti* is a tick-transmitted hematoprotozoan that mainly infects small rodents and can also infect people. Meanwhile, it is the leading cause of human babesiosis in the world (Vannier et al., 2008). Infection of experimental mice is usually self-limiting, and the animals can eventually survive without any clinic symptoms, very similar to the infection in healthy humans (Vannier et al., 2008; Krause, 2019).

With further research on the relationship between parasites and tumors, some protozoan infections are reported to play an antitumor role by activating the host immune response and inhibiting angiogenesis (Callejas et al., 2018). For instance, *Plasmodium yoelii* and *Toxoplasma gondii* could inhibit a variety of tumors in mice, including melanoma and ovarian and lung cancers (Fox et al., 2013; Wang et al., 2020). The two parasites can activate the host immune system and inhibit tumor growth *via* induction of innate and adaptive antitumor immune responses (Chen et al., 2011; Baird et al., 2013a,b). Similarly, *B. microti* infection is also accompanied by complex changes in the host immune response.

The Th1 immune response and macrophages not only are key factors in clearing parasites and controlling infection (Vannier et al., 2004; Elton et al., 2019) but also play an important role in regulating tumor development. Specifically, Th1 cells can produce large amounts of IFN-γ and chemokines to enhance CD8+ T-cell responses, and they can also recruit NK cells and type I macrophages to the tumor site, thus regulating tumor immunity together. Macrophages in the tumor microenvironment exhibit immunosuppressive phenotypes, providing favorable conditions for tumor growth, survival, and angiogenesis, leading to a research hotspot on the strategy of reprogramming tumor-associated macrophages to the M1 type (Nishimura et al., 1999; Pollard, 2004).

Melanoma is a malignant tumor derived from melanocytes (Yde et al., 2018) and also one of the most aggressive and fatal skin tumors (Siegel et al., 2020). Although melanoma accounts for only a small proportion of all skin tumors, the incidence rates are on the rise in recent years (Siegel et al., 2020). Currently, surgery remains the primary treatment for early-stage melanoma (Ross and Gershenwald, 2011), while targeted treatment and immunotherapy are better options for advanced and metastatic melanoma. Targeted therapy generally targets specific mutation sites, while immunotherapy targets the immune system of the body or tumor microenvironment (Franklin et al., 2017). As one of the most immunogenic tumors with high sensitivity to immune regulation, malignant melanoma serves as an excellent model for tumor immunotherapy research (Marzagalli et al., 2019).

Macrophages are a class of cells with a broad role in host defense and immunity, and they are generally classified into classical M1 and alternative M2 macrophages (Mantovani et al., 2002; Gordon and Martinez, 2010). M1 macrophages mainly produce a variety of pro-inflammatory factors, which are important components involved in inflammatory response and antitumor immunity, while M2 macrophages play a role in suppressing inflammation and promoting tumor growth (Chanmee et al., 2014). As an important component of immune cells in the tumor microenvironment, macrophages usually exhibit immunosuppressive phenotypes similar to M2 macrophages and provide favorable conditions for tumor growth, survival, and angiogenesis, so tumor-associated macrophages (TAM) are defined as M2 macrophages in a narrow sense (Pollard, 2004). In melanoma, the enrichment of M1 gene expression signatures showed favorable prognosis, in contrast to poor outcomes for the enrichment of M2 gene expression signatures (Buscher et al., 2017; Ley, 2017). At present, the switch of M2 TAMs back to M1 TAMs has become one of the hotspots in tumor immunotherapy research (Eddy and Chen, 2020).

The purpose of this study was to investigate the effects of *B. microti* infection on melanoma growth and the related mechanism. This study facilitates the understanding of the interaction between *Babesia* infection and melanoma.

## Materials and Methods

### Ethics Statement

During the animal studies, the guidelines for the Regulation of the Administration of Affairs Concerning Experimental Animals of China were followed, and all experiments were approved by the Laboratory Animals Research Centre of Hubei Province and Huazhong Agricultural University (Permit number: HZAUMO-2019-061).

### Mice, Cells, and Parasites

Female 6–8-week-old C57BL/6 mice and BALB/c mice were purchased from the Laboratory Animals Research Centre of Huazhong Agricultural University (Wuhan, China). Murine (B16) and (RAW264.7) cell lines were obtained from China Center for Type Culture Collection (CCTCC). B16 cells were maintained in RPMI 1640 (Gibco, Carlsbad, CA, United States) supplemented with penicillin (80 U/ml), streptomycin (100 U/ml), and 10% FBS in a humidified atmosphere of 5% CO_2_ at 37°C, and RAW264.7 cells were maintained in DMEM (Gibco, Carlsbad, CA, United States) under the same conditions. The *B. microti* strain ATCC^®^ PRA-99TM was obtained from the National Institute of Parasitic Diseases, Chinese Center for Disease Control and Prevention (Shanghai, China), and generally preserved in liquid nitrogen; when large numbers of parasites are needed, they were obtained by infecting mice *in vivo*. The parasites were isolated at parasitemia of 30–40% as determined by Giemsa staining of thin blood smears.

### Tumor Cell Inoculations

A total of 80 C57BL/6 mice were divided into four groups (20 mice per group): (1) naive group: mice with no treatment; (2) B.m group: mice were intraperitoneally infected with 10^7^ *B. microti*-parasitized erythrocytes; (3) B16 group: mice were subcutaneously inoculated with 5 × 10^5^ B16 cells on the right flanks, coupled with intraperitoneal injection of PBS; and (4) B.m+B16 group: mice were subcutaneously inoculated with 5 × 10^5^ B16 cells on the right flanks, coupled with intraperitoneal infection of 10^7^ *B. microti*-parasitized erythrocytes. Parasitemia was determined for B.m and B.m+B16 groups by Giemsa staining of thin blood smears every day. The tumor volume of mice inoculated with B16 cells was measured using a caliper and calculated by the equation: V = (length × width^2^)/2. The length and width were measured using a vernier caliper. In the tumor growth experiment, the mice were euthanized on day 17 based on changes in parasitemia. In the survival experiment, the mice were monitored for 35 days and killed on day 35 post-infection according to tumor size. All the mice were euthanized with ether at the end of the experiment, and all animal experiments were performed once.

### Tissue Dissociation and Flow Cytometry

To test the effects of *B. microti* infection on immune cells in the spleen, a total of 80 mice, with five mice from each of the four groups (naive, B16, B.m, and B.m+B16), were euthanized at four different time points (days 3, 6, 12, and 17). To analyze the effect of *B. microti* infection on the splenocyte of mice, the spleen was sliced into small pieces and filtered through a 70-μm nylon sterile cell strainer into a 50-mL conical tube, followed by washing the cells twice with PBS and removing red blood cells (RBCs) by using RBC lysis (Beyotime Shanghai, China). Briefly, RBC lysis was added as instructed by the manufacturer, followed by mixing and leaving it for 2 min, centrifugation, and removal of the supernatant. After counting, single cell suspension was incubated with an Fc receptor blocker on ice for 10 min, followed by staining dead cells using a Zombie Aqua™ Fixable Viability Kit (Biolegend, United States) and staining the living cells for 30 min in a cell staining buffer with appropriate dilutions of various combinations of the following fluorochrome-conjugated antibodies: anti-CD3 APC/Cy7, anti-CD4 FITC, anti-CD8a PerCP/Cy5.5, anti-NK1.1 APC, and anti-F4/80 PE (Biolegend, United States).

Tumor samples were minced with scissors, followed by incubation with 0.5 mg/ml collagenase IV and 200 U/ml DNase (Sigma-Aldrich, Shanghai, China) in RPMI-1640 medium for 30 min at 37°C, washing the cells twice with PBS, and removing RBCs with RBC lysis. Next, two tubes of cells were prepared for independent staining using the same method as staining the splenocyte, with different antibodies for T cells (anti-CD45 APC/Cy5, anti-CD3 FITC, anti-CD4 PE, anti-CD8a APC) and macrophages (anti-CD45 APC/Cy5, anti-CD11b FITC, anti-F4/80 Brilliant Violet 421, anti-MHC-II APC, anti-CD206 PE). Meanwhile, MHC-II and CD206 were used to mark M1 and M2 macrophages, respectively.

The stained cells were assessed using a CytoFLEX flow cytometer (Beckman Coulter, United States), and data were analyzed using FlowJo software (RRID:SCR_008520, BD Biosciences, United States).

### Cytokine and Nitric Oxide Detection

Mouse sera were collected independently from the four groups (naive, B16, B.m, and B.m+B16) and stored at −80°C for further analysis. RAW264.7 cells were stimulated with a *B. microti* serum-free culture supernatant or normal RBC serum-free culture supernatant for 24 h, followed by collecting the supernatant of RAW264.7 cells. All samples were assayed for TNF-α and IFN-γ by using the mouse ELISA kit (Biolegend, United States) as instructed by the manufacturer. Nitric oxide (NO) was assayed using Griess reagent (Abkkine, China).

### Immunohistochemistry of Tumor Tissue

Mouse tumors were isolated and fixed with 4% paraformaldehyde, followed by preparing paraffin sections, deparaffinization, antigen retrieval, blocking, and incubating tumor tissues with antibodies against mouse CD31 and F4/80 (CST, United States) overnight at 4°C. The following steps were performed as informed in the rabbit two-step detection kit (ZSGB-BIO, Beijing, China). Briefly, after the primary antibody was bound to the antigen in the tissue, HRP-conjugated antibodies were added separately, using DAB as the chromogenic reagent. The images were collected using an optical microscope (Zeiss, Oberkochen, Germany), and the data were analyzed using Image-Pro Plus software 6.0 (RRID:SCR_016879, Media Cybernetics, United States).

### Immunofluorescence Assay

Briefly, RAW264.7 cells were placed on cell culture coverslips at a density of 1 × 10^5^, followed by addition of *B. microti* into the cell culture 12 h later at a 1:20 ratio, incubation independently for 1 and 2 h, discarding the supernatant, and three washes with PBS. Next, the cell culture coverslips were fixed with 4% paraformaldehyde, using both 1:1,000 *B. microti* SA1 polyclonal antibody (Li et al., 2019) and 1:300 mouse beta-actin monoclonal antibody (Affinity Biosciences, Beijing, China) as the primary antibodies, while both 1:1,000 Alexa Fluor 594-conjugated goat anti-mouse IgG and 1:1,000 Alexa Fluor 488-conjugated goat anti-rabbit IgG (Life Technologies, Rockville, United States) as the secondary antibodies. The images were collected using a fluorescence microscope (Zeiss, Oberkochen, Germany).

### Measurement of Macrophage Polarization

To test macrophage polarization *in vivo*, the mice were intraperitoneally injected with 10^7^*B. microti*-infected RBCs or normal RBCs, followed by collecting the peritoneal macrophages separately at 2 and 4 h post-injection.

To test macrophage polarization *in vitro*, RAW264.7 cells were placed on 6-well plates at a density of 5 × 10^5^, followed by adding *B. microti*-infected RBCs or normal RBCs into the culture wells at a ratio of 1:20 and collecting the samples separately after 2 and 4 h of incubation.

To test the stimulation of the *B. microti* culture supernatant on macrophage polarization, RAW264.7 cells were stimulated with *B. microti* serum-free culture supernatant or normal RBC serum-free culture supernatant. After 24 h of treatment, RAW264.7 cell samples were collected, followed by detecting the concentrations of TNF-α and NO in the culture supernatant, as described in the section of *Cytokine and nitric oxide detection*.

RNA was extracted from all the collected samples, followed by reverse transcription into cDNA, and the expression of iNOS, IL-6, and TNF-α was detected and analyzed by qPCR. qPCR was conducted using ChamQ Universal SYBR qPCR Master Mix (Vazyme, China) under the conditions of an initial denaturation step of 95°C for 30 s, followed by 40 cycles of 10 s at 95°C and 10 s at 60°C. After completion of the qPCR amplification, a melting curve analysis was run to evaluate the amplification specificity.

To test the effect of the *B. microti* culture supernatant on M2 macrophage polarization, RAW264.7 cells were cultured with 20 ng/mL IL-4 for 24 h, followed by inducing the conversion of macrophages into the M2 type, adding the *B. microti* culture supernatant into the culture wells, and incubation for 24 h. The mRNA expression levels of M1 markers (iNOS, IL-6, TNF-α) and M2 markers (CD206 and IL-10) in RAW264.7 cells, and the concentrations of TNF-α and NO in the culture supernatant of RAW264.7 cells were detected as described previously.

The primers for quantitative PCR are shown in Supplementary Table 1.

### Statistical Analysis

Animal experiments were performed only once, and other experiments *in vitro* were repeated at least twice with a similar result. The results are expressed as mean ± standard deviation (SD). All data were analyzed using GraphPad Prism 7.0 (GraphPad Software, RRID:SCR_002798, United States) by two-way analysis of variance (ANOVA) (dynamics of parasitemia and the growth kinetics of subcutaneous tumor), log-rank (Mantel–Cox) test (survival experiment), and unpaired Student’s *t*-test (the other data). Significant differences between groups were determined at *p ≤ 0.0332, ^**^p ≤ 0.0021, ^***^p ≤ 0.0002, and ^****^p ≤ 0.0001.

## Results

### *Babesia microti* Infection Inhibits Mouse Melanoma Growth

**FIGURE 1:**
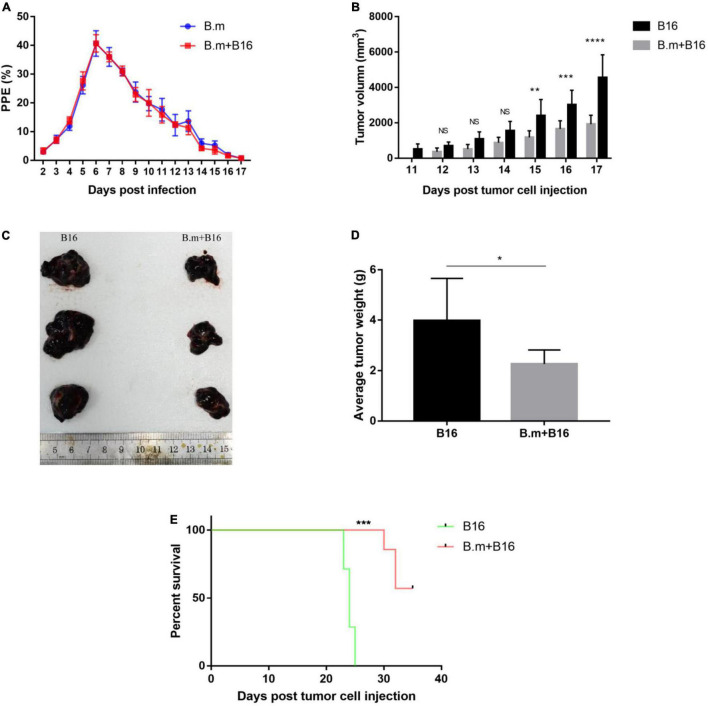
Effects of *B. microti* infection on melanoma growth and survival time of tumor-bearing mice. **(A)** Dynamics of parasitemia measured by blood smear in *B. microti*-infected mice. **(B)** Growth kinetics of subcutaneous tumor in *B. microti* infected and uninfected tumor-bearing mice. **(C)** Tumor size of part of mice. **(D)** Weight of subcutaneous tumor at day 17 post-tumor cell injection. **(E)** Survival curve of tumor-bearing mice from day 0 to 35 post-tumor cell injection. Error bars are SD; *p ≤ 0.0332, **p ≤ 0.0021, ***p ≤ 0.0002, ****p ≤ 0.0001. There were seven mice in each group.

The interaction between *B. microti* infection and the growth of melanoma cells was investigated by inoculating mice with melanoma cells and/or *B. microti* simultaneously and monitoring the parasitemia and tumor development. In Figure 1A, blood smear showed that melanoma had no effect on parasitemia, with a similar parasitemia pattern for both B.m and B.m+B16 groups, and their respective parasitemia were 2.93 and 3.32% on day 2 and reached the peak (40.66 and 40.72%) on day 6 post-infection.

In Figures 1B–D, *B. microti* infection was seen to inhibit the size of subcutaneous tumors. The B16 group developed measurable tumors on day 11, in contrast to no measurable tumors for the B.m+B16 group until day 12 (Figure 1B). On day 15 post-infection, the tumor volume was significantly (*p* ≤ 0.0021) smaller (∼51%) in the B.m+B16 group than in the B16 group, and the difference was more (∼58%) significant on day 17 (*p* ≤ 0.0001). The subcutaneous tumors were isolated on day 17, and the tumor size and weight were significantly (*p* ≤ 0.0332) smaller (∼44%) in the B.m+B16 group than in the B16 group (Figures 1C,D). *B. microti* infection also significantly (*p* ≤ 0.0002) prolonged the survival period of tumor-bearing mice, with no survivors in the B16 group on day 25, in contrast to the B.m+B16 group, where all mice survived at day 30 post-infection. After the survival experiment on day 35 post-infection, 57.14% (4/7) of the infected mice still survived (Figure 1E).

### *Babesia microti* Infection Alters the Spleen Immune Environment

**FIGURE 2:**
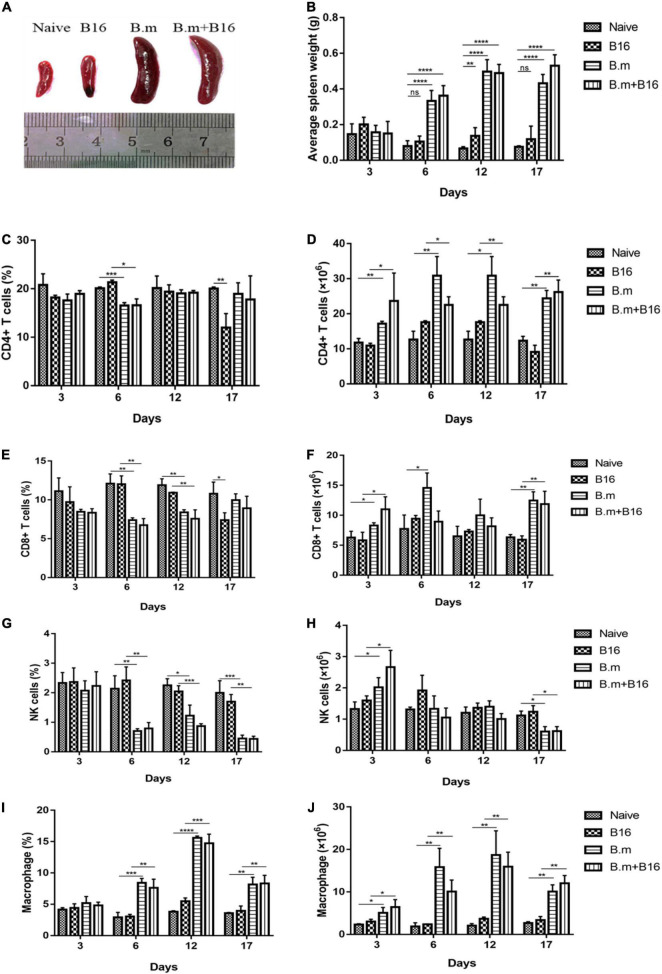
Effects of *B. microti* infection on spleen morphology and splenocytes in mice. **(A)** Images of spleens in the four groups, with the spleen size being significantly increased in *B. microti*-infected mice vs. uninfected mice. **(B)** Changes of spleen weight of mice from day 3 to 17 post-tumor cell injection, with the weight of the spleen being significantly increased in the infection process. **(C,D)** Proportion and number of CD4+ T cells in the spleen of each group from day 3 to 17 post-tumor cell injection. **(E,F)** Proportion and number of CD8+ T cells in the spleen of each group from day 3 to 17 post-tumor cell injection. **(G,H)** Proportion and number of NK cells in the spleen of each group from day 3 to 17 post-tumor cell injection. **(I,J)** Proportion and number of macrophages in the spleen of each group from day 3 to 17 post-tumor cell injection. Error bars are SD; *p ≤ 0.0332, **p ≤ 0.0021, ***p ≤ 0.0002, ****p ≤ 0.0001, ns: no significance. There were 5 mice in each group.

*B. microti* infection changed the size and weight of the spleen in mice (Figure 2A). Both B.m (0.4971 g) and B.m+B16 (0.4897 g) groups showed significantly (*p* ≤ 0.0021) enlarged and swollen spleens from day 6 post-infection compared to naive (0.1372 g) and B16 (0.07996 g) groups (Figure 2B).

The effect of *B. microti* infection on the mouse immune system was evaluated by analyzing the proportion and number of T cells, NK cells, and macrophages in the spleen on days 3, 6, 12, and 17 post-infection. Despite no significant (*p* > 0.0332) change in the proportion of CD4+ T cells in the spleen of all groups on day 3, B.m (16.57%), and B.m+B16 (16.6%) groups showed a transient decrease in the proportion of CD4+ T cells as compared to the naive (20.1%) and B16 (21.33%) groups on day 6, followed by a return to normal on day 12 (Figure 2C). The number of CD4+ T cells in the spleen significantly (*p* ≤ 0.0332) increased in B.m and B.m+B16 groups relative to naive and B16 groups during the entire infection period. The number of CD4+ T cells in the B.m+B16 group was about three times that of the B16 group on day 17 (Figure 2D). On day 6, B.m (7.39%) and B.m+B16 (6.75%) groups showed a decrease in the proportion of CD8+ T cells in the spleen when compared with the naive (12.1%) and B16 (12.03%) groups, followed by a gradual return to normal on day 17. Additionally, B.m and B.m+B16 groups showed no significant differences in the proportion of CD8+ T cells on days 17 and 3 (Figure 2E), but an increase (*p* ≤ 0.0332) in the number of CD8+ T cells in the spleen when compared to the naive and B16 groups during the infection period, except for day 12. The number of CD8+ T cells in the B.m+B16 group was doubled than the B16 group on day 17 approximately (Figure 2F). Moreover, B.m (0.7%) and B.m+B16 (0.79%) groups were significantly (*p* ≤ 0.0021) lower than the naive (2.14%) and B16 (2.42%) groups in the proportion of NK cells in the spleen on day 6, and further decreased (*p* ≤ 0.0021) [B.m (0.45%) and B.m+B16 (0.44%)] on day 17 (Figure 2G). Meanwhile, B.m (0.6006 × 10^6^) and B.m+B16 (0.6175 × 10^6^) groups were also significantly (*p* ≤ 0.0332) lower than naive (1.1146 × 10^6^) and B16 (1.239 × 10^6^) groups in the number of NK cells on day 17 (Figure 2H). On day 6, compared with naive (2.92%) and B16 (3.04%) groups, B.m (8.41%) and B.m+B16 (7.6%) groups significantly (*p* ≤ 0.0021) increased in the proportion of spleen macrophages and reached the peak (B.m 15.6% and B.m+B16 14.73%) on day 12 (Figure 2I). In Figure 2J, the number of macrophages was seen to be significantly (*p* ≤ 0.0332) increased in B.m and B.m+B16 groups relative to naive and B16 groups during the whole infection process. The number of macrophages in the B.m+B16 group was more than four times as many as the B16 group on day 12. These results indicated that *B. microti* infection can affect the immune system of tumor-bearing mice, especially macrophages.

### *Babesia microti* Infection Does Not Induce a Strong Fluctuation in Concentration of IFN-γ and TNF-α

**FIGURE 3:**
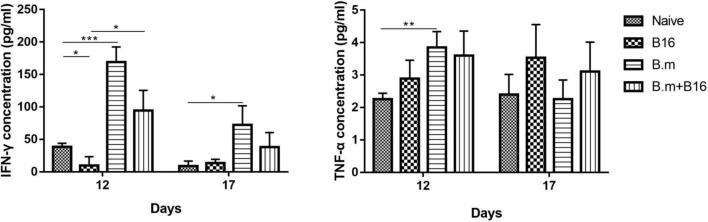
Effects of *B. microti* infection on the concentration of IFN-γ and TNF-α in mouse serum on days 12 and 17 post-tumor cell injection. Mice serums of the four groups were collected on days 12 and 17 post-tumor cell injection, respectively, and the concentration of IFN-γ and TNF-α in serum was detected by using the ELISA kit. Error bars are SD; *p ≤ 0.0332,**p ≤ 0.0021, ***p ≤ 0.0002. There were five mice in each group.

Figure 3 shows the effects of *B. microti* infection on the concentration of IFN-γ and TNF-α in mice serum on days 12 and 17 post-tumor cell injection. On day 12, IFN-γ concentration was lower (∼74%, *p* ≤ 0.0332) in the B16 group than in the naive group, while significantly (*p* ≤ 0.0332) higher in B.m (169.1 pg/mL) and B.m+B16 (94.19 pg/mL) groups than in naive (38.62 pg/mL) and B16 (9.897 pg/mL) groups. On day 17, the IFN-γ concentration decreased in B.m (72.46 pg/mL) and B.m+B16 (38.22 pg/mL) groups but was still higher than that in naive (9.128 pg/mL) and B16 (13.74 pg/mL) groups. On day 17, no significant difference was observed among the four groups in TNF-α concentration, but on day 12, the B.m group (3.851 pg/mL) was significantly (*p* ≤ 0.0021) higher than naive (2.255 pg/mL) in TNF-α concentration.

### *Babesia microti* Infection Inhibits Tumor Angiogenesis and Alters the Tumor Immune Microenvironment

**FIGURE 4:**
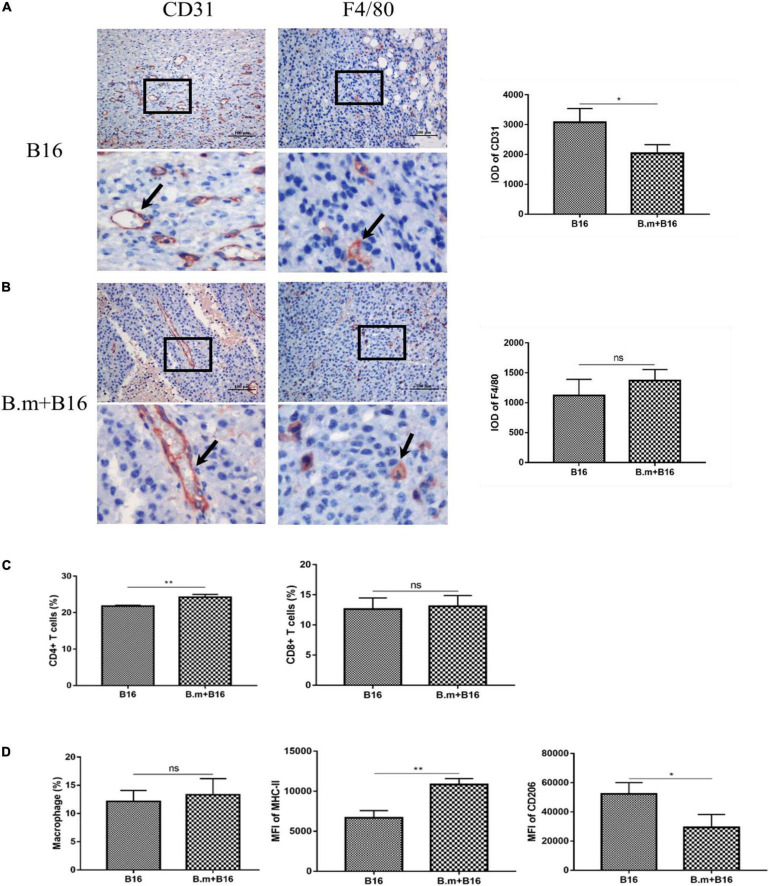
Effects of *B. microti* infection on tumor angiogenesis and tumor immune microenvironment. **(A,B)** Immunohistochemical analysis of the expression of CD31 and F4/80 in mouse tumor tissues, with positive staining indicated by arrows. IOD of positive staining was statistically analyzed using IPP software (right panel). **(C)** Proportion of CD4+ and CD8+ T cells in tumors. **(D)** Proportion of macrophages in tumors (left panel), and the median fluorescence intensity (MFI) of MHC-II and CD206 in macrophages (middle and right panels). Error bars are SD; *p ≤ 0.0332, **p ≤ 0.0021, ns: no significance.

CD31 is a well-known marker of endothelial cells commonly used to evaluate tumor angiogenesis, which can promote the growth and metastasis of tumors, so we detected the expression of CD31 in tumors. Figure 4 shows the effects of *B. microti* infection on tumor angiogenesis and tumor immune microenvironment. In Figure 4A, the B.m+B16 group was seen to be significantly (*p* ≤ 0.0332) lower (∼34%) than the B16 group in the expression of CD31 in tumors, suggesting that *B. microti* infection could inhibit angiogenesis in tumors (Figure 4A). The expression of F4/80 in tumor was also detected to evaluate the number of tumor-associated macrophages. In Figure 4B, B.m+B16 and B16 groups were seen to have no significant (*p* = 0.3003) difference in the number of macrophages.

The proportion of T cells and macrophages in the subcutaneous tumor was also measured on day 17 post-infection. In Figure 4C, compared with the B16 group (21.8%), the B.m+B16 group (24.225%) showed a significant (*p* ≤ 0.0021) increase in the proportion of CD4+ T cells in the tumors, while no significant (*p >* 0.0332) difference in the proportion of CD8+ T cells. The gating strategy of tumor-associated macrophages is shown in Supplementary Figure 1. In Figure 4D, B16 and B.m+B16 groups showed no significant (*p >* 0.0332) difference in the proportion of macrophages in the tumor microenvironment, but the B.m+B16 group exhibited a significant (*p* ≤ 0.0021) increase (∼ 63%) in the median fluorescence intensity (MFI) of MHC-II and a decrease (∼ 44%, *p* ≤ 0.0332) in the MFI of CD206 relative to the B16 group.

### Macrophages Take Up *Babesia microti* and Are Activated by Parasites

**FIGURE 5:**
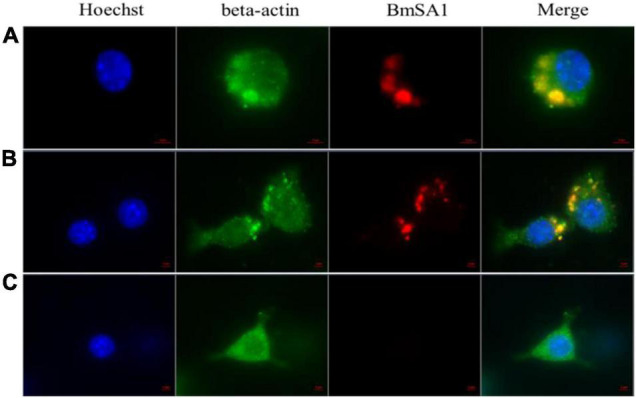
Immunolocalization of *B. microti* internalized in macrophages. **(A)** Incubation of *B. microti*-infected RBCs with macrophages for 1 h. **(B)** Incubation of *B. microti*-infected RBCs with macrophages for 2 h. **(C)** Negative control: incubation of normal RBCs with macrophages. Scale bar = 2 μm.

Figure 5 shows the immunolocalization of *B. microti* internalized in macrophages. In Figures 5A,B, parasites were shown to be localized in the cytoplasm of macrophages after incubating *B. microti* with macrophages for 1 and 2 h. Figure 5C is a negative control without *B. microti*. IFA results indicated that macrophages could take up *B. microti*.

**FIGURE 6:**
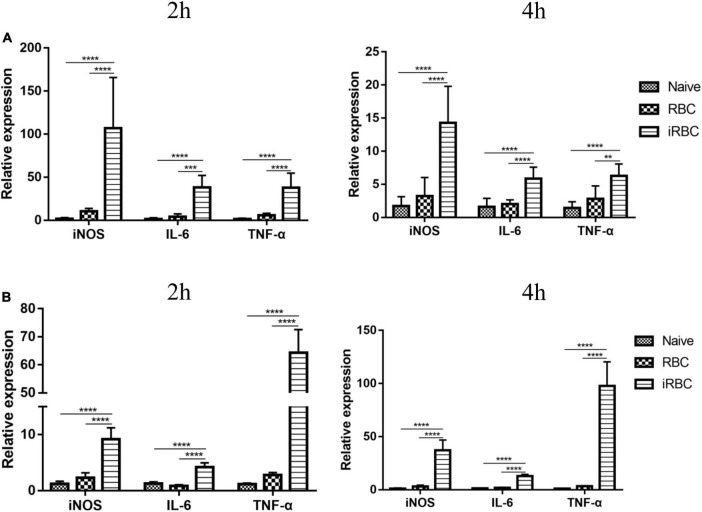
Effects of *B. microti* on macrophage polarization. **(A)** qPCR analysis of the effects of *B. microti* on the expression levels of iNOS, IL-6, and TNF-α in peritoneal macrophages; mice were intraperitoneally injected with *B. microti*, followed by collecting peritoneal macrophages separately at 2 and 4 h post-injection. **(B)** qPCR analysis of the effects of *B. microti* on the expression levels of iNOS, IL-6, and TNF-α in RAW264.7 macrophage cells; *B. microti*-infected RBCs were incubated with RAW264.7 cells for 2 and 4 h, respectively. Error bars are SD; **p ≤ 0.0021, ***p ≤ 0.0002, ****p ≤ 0.0001.

Flow cytometric analysis of tumors revealed changes in the polarization of macrophages in the tumors of the B.m+B16 group, so the effects of *B. microti* infection on macrophage polarization *in vivo* and *in vitro* were tested. Figure 6 shows the effects of *B. microti* on macrophage polarization. In Figure 6A, *B. microti* infection was seen to significantly (*p* ≤ 0.0001) increase the mRNA expression of iNOS (∼ 60 times), IL-6 (∼ 22 times), and TNF-α (∼ 25 times) in peritoneal macrophages relative to the naive group at 2 h and ∼ 7 times, 2.6 times, and 3 times at 4 h, respectively, post-tumor cell infection. Similar patterns were also observed *in vitro*, where *B. microti* showed a significant (*p* ≤ 0.0001) increase in the mRNA expression of iNOS (∼ 6.5 times), IL-6 (∼ 2.3 times), and TNF-α (∼ 55 times) in RAW264.7 cells at 2 h and ∼ 29 times, 9 times, and 84 times at 4 h, respectively (Figure 6B). These results suggested that *B. microti* could stimulate the expression of pro-inflammatory genes in macrophages and induce macrophage polarization in a short time.

### *Babesia microti* Culture Supernatant Can Induce Macrophage Polarization *in vitro*

**FIGURE 7:**
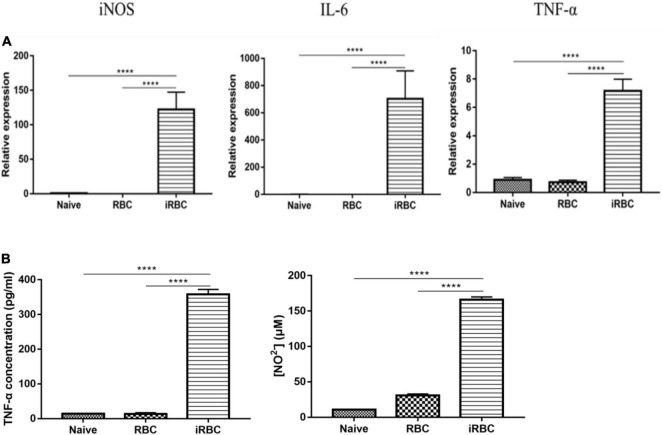
Effects of *B. microti* culture supernatant on macrophage polarization. **(A)** qPCR analysis of the effects of *B. microti* culture supernatant on the expression levels of iNOS, IL-6, and TNF-α in RAW264.7 macrophage cells; the serum-free culture supernatant of *B. microti* was collected and incubated with RAW264.7 cells for 24 h. **(B)** Concentration of TNF-α and NO^2–^ in RAW264.7 cell culture supernatant as detected by using the ELISA kit and Griess reagent, respectively. Error bars are SD; ****p ≤ 0.0001.

The effects of the *B. microti* serum-free culture supernatant on macrophage polarization were investigated by incubating RAW264.7 cells with the *B. microti* serum-free culture supernatant for 24 h and analyzing the mRNA expression of iNOS, IL-6, and TNF-α in RAW264.7 cells and the concentration of TNF-α and NO in the culture supernatant of RAW264.7 cells. Figure 7 presents the effects of the *B. microti* culture supernatant on macrophage polarization. In Figure 7A, the *B. microti* culture supernatant was seen to significantly (*p* ≤ 0.0001) increase the mRNA expression of iNOS (∼ 166 times), IL-6 (∼1,225 times), and TNF-α (∼ 7 times) in RAW264.7 cells, and the concentration of TNF-α (∼ 25 times) and NO (∼ 4 times) was also significantly (*p* ≤ 0.0001) increased (Figure 7B). These results indicated that the *B. microti* serum-free culture supernatant can also induce macrophage polarization.

### *Babesia microti* Culture Supernatant Can Induce Repolarization of M2 Macrophage to M1 Type *in vitro*

**FIGURE 8:**
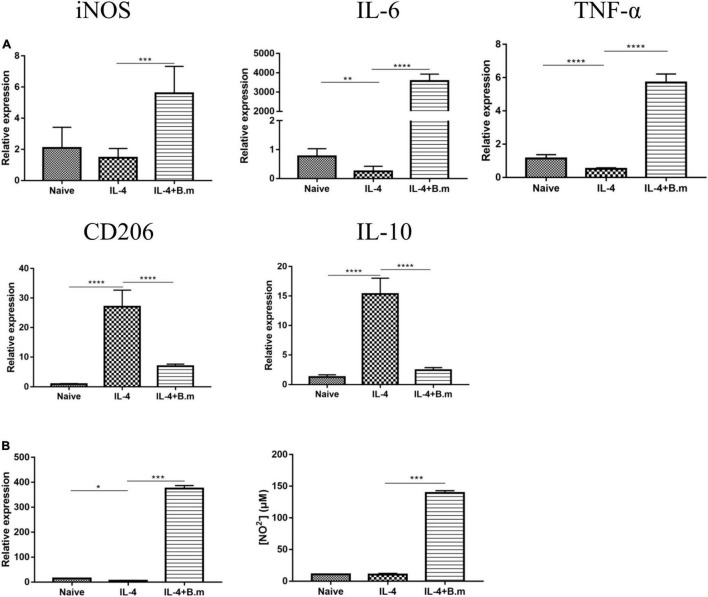
Effects of *B. microti* culture supernatant on the polarization of M2 macrophage to the M1 phenotype. **(A)** qPCR analysis of the effects of *B. microti* culture supernatant on the expression of iNOS, IL-6, TNF-α, CD206, and IL-10 in RAW264.7 cells treated with IL-4; RAW264.7 cells were cultured with mouse recombinant IL-4 for 24 h and then incubated with the *B. microti* culture supernatant for 24 h. **(B)** Concentrations of TNF-α and NO^2–^ in the cell culture supernatant as detected by using the ELISA kit and Griess reagent, respectively. Error bars are SD; *p ≤ 0.0332, **p ≤ 0.0021, ***p ≤ 0.0002, ****p ≤ 0.0001.

The effects of the *B. microti* culture supernatant on the polarization of M2 macrophages to the M1 phenotype are presented in Figure 8. In Figure 8A, it was shown that compared with the naive group, the IL-4 group showed a significant (*p* ≤ 0.0021) decrease in the expression of M1 macrophage markers, for example, iNOS (∼30%), IL-6 (∼68%), and TNF-α (∼55%), while an increase (*p* ≤ 0.0001) in the expression of M2 macrophage markers, for example, CD206 (∼30 times) and IL-10 (∼11 times). After M2 macrophages were treated with the *B. microti* culture supernatant, the IL-4+B.m group showed a significant (*p* ≤ 0.0002) increase in the expression of iNOS (∼3 times), IL-6 (∼14,473 times), and TNF-α (∼10 times), while a notable decrease (*p* ≤ 0.0001) in the expression of CD206 (∼3 times) and IL-10 (∼5 times) (Figure 8A). After *B. microti* culture supernatant treatment, the concentration of TNF-α (∼65 times) and NO (∼12 times) was also significantly (*p* ≤ 0.0002) increased (Figure 8B). These results indicated that the *B. microti* serum-free culture supernatant could induce the repolarization of M2 macrophages to M1 type.

## Discussion

Since the discovery of the therapeutic effect of Coley toxin on cancer, microbial antitumor therapy has received more attention and has been widely studied (Kienle, 2012). Studies have found that Bacillus Calmette-Guérin (BCG), intestinal probiotics, and other bacteria can indirectly play an antitumor effect by stimulating the innate immune response of the body (So et al., 2017; Eslami et al., 2019; Li et al., 2021; Sfakianos et al., 2021). Oncolytic viruses were reported to selectively replicate in and kill cancer cells without harming normal tissues, leading to the approval of several oncolytic viral therapies for the treatment of advanced tumors (Fukuhara et al., 2016; Ma et al., 2018). In recent years, several studies have revealed a close relationship between parasites and tumors. Some parasites, such as *Schistosoma* and *Clonorchis sinensis*, are important causes of cancer, while some parasites, such as *Plasmodium yoelii* and *Toxoplasma gondii*, have been found to have antitumor effects (Won et al., 2014; Fox et al., 2016; Liao et al., 2018; Hamid, 2019; Wang et al., 2020). Protozoal infection can stimulate the immune response of the host.

In this study, we evaluated the effects of *B. microti* infection on B16 melanoma growth. B16 cells are spontaneous tumor cells from C57BL/6 mice, which are widely used as a melanoma experimental model (Overwijk and Restifo, 2001). In blood smear analysis, melanoma was shown to have no effect on parasitemia. The measurable subcutaneous solid tumor appeared on day 11 after inoculation of B16 cells in mice, while on day 12 for the B.m+B16 group. On day 17, the tumor weight and volume were significantly smaller (∼ 44 and 58%, respectively) in the B.m+B16 group than in the B16 group. In the survival assay, all mice in the B16 group died on day 25, while all mice in the B.m+B16 group survived at day 30, and 57.14% (4/7) of them still survived on day 35 post-tumor cell infection when all the mice were killed. In immunohistochemical assays, *B. microti* infection was shown to inhibit tumor angiogenesis. All these results indicated that *B. microti* infection could effectively inhibit the growth of melanoma cells or tumor angiogenesis and increase the survival time of tumor-bearing mice.

The spleen, the largest immune organ in the body, plays an important role in the clearance of *B. microti* infection. In this study, the spleen of all *B. microti*-infected mice was shown to be significantly enlarged, and the immune system was activated. In the early infection stage, the B.m+B16 group showed no significant change in the proportion of CD4+ T cells, CD8+ T cells, NK cells, and macrophages in the spleen compared to the B16 group. However, there was a slight increase in the number of these four kinds of cells mentioned earlier. Then the proportion of CD4+ T cells and CD8+ T cells began to decrease on day 6, probably due to the depletion of cells during the clearance phase of infection. As the infection was gradually cleared, the proportion of cells returned to normal on day 17. The number of CD4+ T cells and CD8+ T cells increased, thus enlarging the spleen and changing the organ’s architecture. The proportion and number of macrophages kept a steady increase in the middle and late stages of infection. However, whether immune cells from the spleen play a role in tumor suppression needs to be further confirmed in future studies. Analysis of immune cells in tumor tissues showed that the proportion of CD4+ T cells was higher in the tumors of *B. microti*-infected mice than in the uninfected mice. Although the proportion of macrophages remained unchanged, the infected mice showed an increase in the expression of MHC-II in macrophages and a decrease in the expression of CD206. These results suggested that *B. microti* infection can recruit CD4+ T cells into the microenvironment, increasing the proportion of M1 macrophages and decreasing the proportion of M2 macrophages. The tumor is rich in blood vessels, and the *B. microti*-infected RBCs would follow the bloodstream to the blood vessels inside the tumor. We supposed that the body may recruit immune cells into the tumor to clear parasites in the blood vessels. Despite intratumoral macrophage phenotypes were analyzed in this study, macrophage depletion assays are needed to confirm the key role and mechanism of macrophages in *B. microti* antitumor process.

Moreover, the *B. microti*-infected mice had significantly higher serum IFN-γ concentration than the uninfected mice on day 12 post-tumor cell infection, but with no significant difference between infected mice and uninfected mice on day 17. Meanwhile, the two groups showed no significant difference in the serum TNF-α concentration. Previous studies have shown that *B. microti* infection did not cause excessive “inflammatory cytokine storm,” which may partially account for why *B. microti* infection did not cause an excessive pathological response in mice (Skariah et al., 2017), because mice are confirmed to be one of the natural reservoirs of *B. microti*.

CD4+ T cells and macrophages have been shown to play an important role in clearing *B. microti* infection (Terkawi et al., 2015; Djokic et al., 2018), as well as in antitumor immunity. The tumor microenvironment is immunosuppressed, and *B. microti* infection can stimulate the activation of the immune system. After observing the inhibitory effect of *B. microti* infection on tumor growth, we assumed that *B. microti* might exert an antitumor effect by changing the immune state of the tumor microenvironment, and this change might be non-specific, which needed to be confirmed by further experiments. CD4+ T cells can affect tumor growth either directly or indirectly by secreting a variety of cytokines. In this study, we found that a larger number and proportion of CD4+T cells were produced in the spleen of *B. microti*-infected tumor-bearing mice for clearance of infection, suggesting that *B. microti* infection may activate the immune system of tumor-bearing mice and cause the body to produce large amounts of CD4+T cells, thereby inhibiting tumor growth (Nishimura et al., 1999).

Macrophages are an important component of immune cells in the tumor microenvironment, which exhibit immunosuppressive phenotypes similar to M2-type macrophages, providing favorable conditions for tumor growth, survival, and angiogenesis (Pollard, 2004). However, M1 macrophages can release a variety of pro-inflammatory factors, immune activators, and chemokines, playing an antitumor role through acute pro-inflammatory response, immune activation, and cytophagocytosis. In recent years, TAMs have become a popular target in cancer immunotherapy because of their ability to regulate the functions of various immune cells. The strategy of reprogramming M2 TAMs to M1 phenotypes using toll-like receptor agonists to produce various pro-inflammatory factors has achieved good efficacy in mouse models (Tran et al., 2019). Several studies have shown that macrophages are one of the important factors in the clearance of *B. microti*, and macrophages can take up parasites (Boucher et al., 2018; Winkel et al., 2020). In this study, we observed that macrophages can take up *B. microti in vitro*, but due to the extremely small number of macrophages in tumor tissue, we could not directly prove whether macrophages in tumor phagocytize *B. microti*. Meanwhile, no report is available regarding whether other phagocytic cells can take up *B. microti*, such as dendritic cells. Additionally, we also found that both infected erythrocytes and *B. microti* culture supernatants could significantly induce the mRNA expression of iNOS, IL-6, and TNF-α in macrophages and activate macrophages. The *B. microti* culture supernatant could also convert IL-4-induced M2 macrophages to the M1 type. Previous studies also mentioned that *Babesia* could produce a large number of antigens able to be secreted outside the parasite (Li et al., 2019), and a variety of proteins were secreted from the *in vitro* culture supernatant of *B. microti* (Li et al., 2019). Glycosylphosphatidylinositol (GPI) anchors, the most abundant pathogen-associated molecular patterns (PAMPs) identified in protozoa, are composed of a glycan core and a lipid component, and GPI anchors of *Plasmodium* were reported to induce pro-inflammatory response by stimulating macrophages to produce TNF-α, IL-1β, IL-6, IL-12, and NO (Zhu et al., 2005), providing a reference for us to study the function of GPI anchors of *B. microti*. In this study, both *B. microti*-infected erythrocytes and culture supernatant can stimulate the production of pro-inflammatory factors in macrophages, suggesting that the GPI anchor of *B. microti* may be one of the important factors inducing the activation of macrophages. GPI protein not only exists on the surface of the parasite but also can be secreted outside of the parasite (Li et al., 2019). However, whether the GPI anchor of *B. microti* can stimulate macrophage activation has not been demonstrated, and the specific mechanism for macrophage activation by *B. microti* needs to be verified in further studies.

## Conclusion

In this study, we found that *B. microti* infection can effectively inhibit the growth of melanoma cells and prolong the survival time of tumor-bearing mice by stimulating the immune system, especially increasing the number of CD4+ T cells and macrophages. Meanwhile, *B. microti* can also induce the activation of macrophages and the conversion of M2 macrophages to the M1 type.

## Data Availability Statement

The original contributions presented in the study are included in the article/Supplementary Material, further inquiries can be directed to the corresponding author/s.

## Ethics Statement

The animal study was reviewed and approved by the Laboratory Animals Research Centre of Hubei Province and Huazhong Agricultural University.

## Author Contributions

XS, ZN, WL, HZ, ZH, YX, and HD performed the experiments. XS, YZ, FL, and SW participated in the data analysis. LH and JZ edited the manuscript. All authors contributed to the article and approved the submitted version.

## Conflict of Interest

The authors declare that the research was conducted in the absence of any commercial or financial relationships that could be construed as a potential conflict of interest.

## Publisher’s Note

All claims expressed in this article are solely those of the authors and do not necessarily represent those of their affiliated organizations, or those of the publisher, the editors and the reviewers. Any product that may be evaluated in this article, or claim that may be made by its manufacturer, is not guaranteed or endorsed by the publisher.

## Supplementary Material

Supplementary Figure 1 Gating strategy for tumor-associated macrophages. Leukocytes from living cells were labeled with CD45, and macrophages from leukocytes were labeled with F4/80 and CD11b, followed by using MHCII and CD206 to label M1 and M2 macrophages, respectively.
