# Supplementary material for: Babesia microti Infection Inhibits Melanoma Growth by Activating Macrophages in Mice
Source: Front Microbiol. 2022 Jun 22;13:862894. doi: 10.3389/fmicb.2022.862894 (PMC9257138; doi:10.3389/fmicb.2022.862894)
Supplement: Supplementary file 1 [file Table_1.DOCX]

Table S1 The primers for quantitative PCR

| Primer | Primer sequence (5’-3’) |
| --- | --- |
| GAPDH-F | AGGTCGGTGTGAACGGATTTG |
| GAPDH-R | GGGGTCGTTGATGGCAACA |
| iNOS-F | AGAGTCACCAAAATGGCTCCC |
| iNOS-R | TACTGTGGACGGGTCGATGT |
| IL-6-F | TCGGAGGCTTAATTACACATGTTCT |
| IL-6-R | GCAAGTGCATCATCGTTGTTCATA |
| TNF-α-F | ATGGCCTCCCTCTCATCAGT |
| TNF-α-R | TGGTTTGCTACGACGTGGG |
| CD206-F | AAGGTTCGGGATTGTGGAGC |
| CD206-R | TTTGCATTGCCCAGTAAGGAGT |
| IL-10-F | ACTGCTAACCGACTCCTTAATGC |
| IL-10-R | CTGGGGCATCACTTCTACCA |
