# Supplementary figures and images for: Babesia microti Infection Inhibits Melanoma Growth by Activating Macrophages in Mice
Source: Front Microbiol. 2022 Jun 22;13:862894. doi: 10.3389/fmicb.2022.862894 (PMC9257138; doi:10.3389/fmicb.2022.862894)

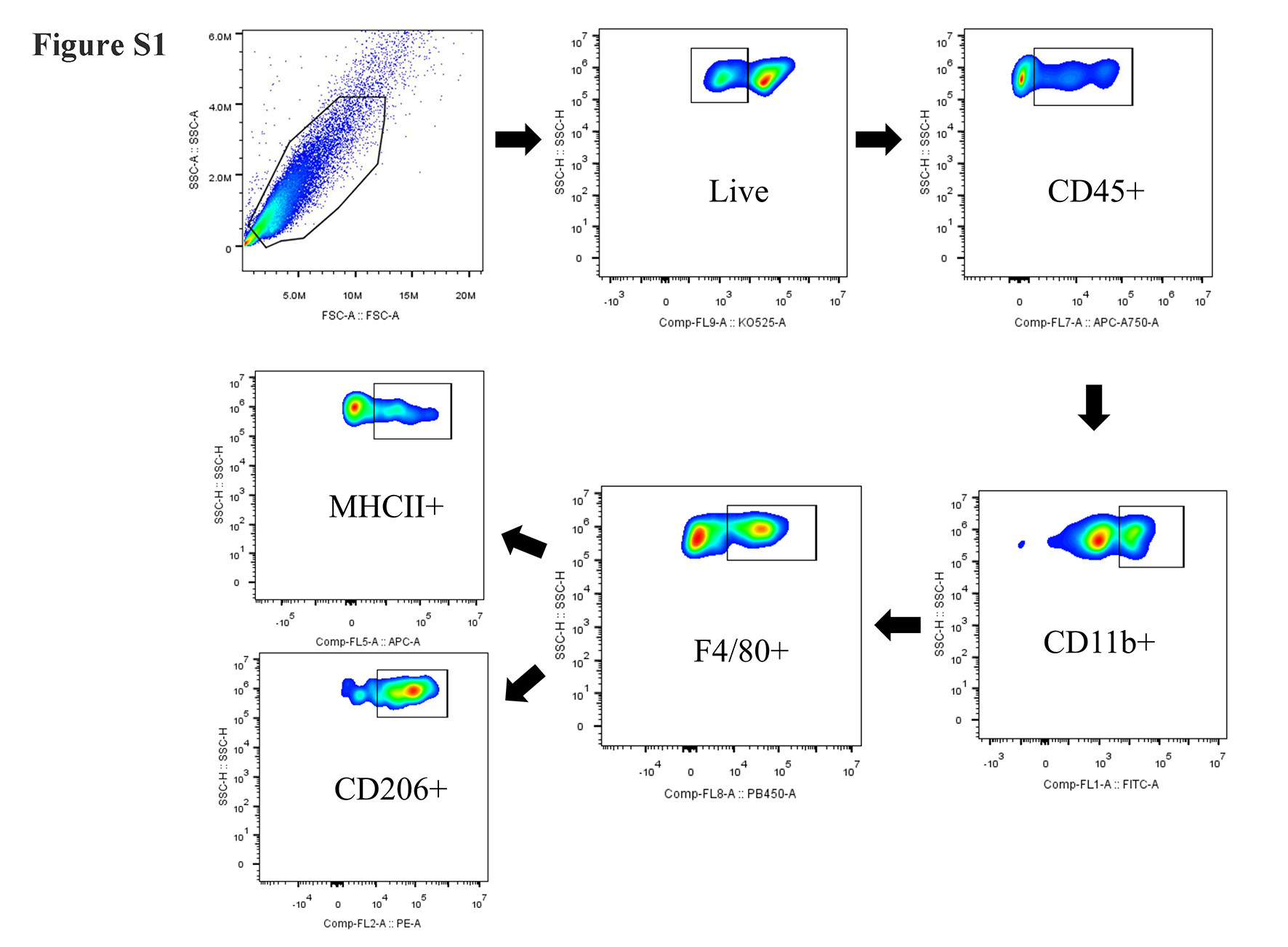

Supplement: Supplementary Figure 1 — Gating strategy for tumor-associated macrophages. Leukocytes from living cells were labeled with CD45, and macrophages from leukocytes were labeled with F4/80 and CD11b, followed by using MHCII and CD206 to label M1 and M2 macrophages, respectively. [file Image_1.TIF]
